# Laparoscopic Repair of Right Paraduodenal Hernia in Adult Patients: Case Report and Literature Review

**DOI:** 10.1155/2018/9691689

**Published:** 2018-10-15

**Authors:** Tomoko Takagishi, Yuta Niimi, Goshi Matsuki, Shinta Nagano, Junsuke Hinami, Masaaki Kajiwara, Kiyoshi Kaneko, Yoshihiro Kubota, Osamu Nakai

**Affiliations:** Department of Surgery, Uji Tokushukai Medical Center, Uji, Kyoto 611-0041, Japan

## Abstract

A 56-year-old Japanese female presented with vomiting, nausea, and abdominal pain after excessive drinking and eating. Abdominal computed tomography showed an encapsulated circumscribed cluster of jejunal loops in the right upper quadrant. She was diagnosed with a strangulated intestinal obstruction caused by right paraduodenal hernia (PDH) and underwent an emergency laparoscopic repair. A view through the endoscope showed the right PDH, which was encapsulated under the mesocolon. Most of the small bowel was entrapped and adhered inside the sac, requiring careful adhesiolysis. The hernia orifice was expanded to a sufficient degree, and the strangulation was relieved, avoiding the need of resecting the small intestine. Recovery was uneventful, and the patient remains free of symptoms 3 years after surgery. Findings in a total of 29 patients (including this report) who underwent laparoscopic repair of right or left PDHs in Japan are discussed.

## 1. Introduction

Recognizing late-onset complications of congenital intestinal malrotation in adult patients is important [1]. Paraduodenal hernia (PDH) is the most common type of intra-abdominal hernia associated with congenital errors of rotation of the midgut, in which duodenal recesses, particularly paraduodenal recess, play a role [2]. PDH accounts for 53% of internal hernias, but only 0.2% to 5.8% of intestinal obstructions, and occurs in both adults and children [3]. Patients with PDH present with various symptoms, including those of acute or chronic obstruction and intermittent abdominal pain associated with nausea and vomiting [1], and some patients may present with a history of recurrent intestinal obstruction since childhood [4]. Two variants of PDH are known, right-sided and left-sided, with the latter being more common. Surgical repair of PDH has shifted dramatically from open laparotomy to laparoscopic procedures [5]. The clinical features of right-sided PDH have been described [4, 6–8], and advances in laparoscopic surgical techniques have permitted the safe and effective laparoscopic treatment of right-sided PDH [4, 6–13]. This report describes the successful laparoscopic repair of a right-sided PDH in an adult patient. This study also reviews Japanese experiences with laparoscopic repair of PDH, comparing clinical features and surgical results in adult patients with right- or left-sided PDH.

## 2. Case Report

**Figure 1:**
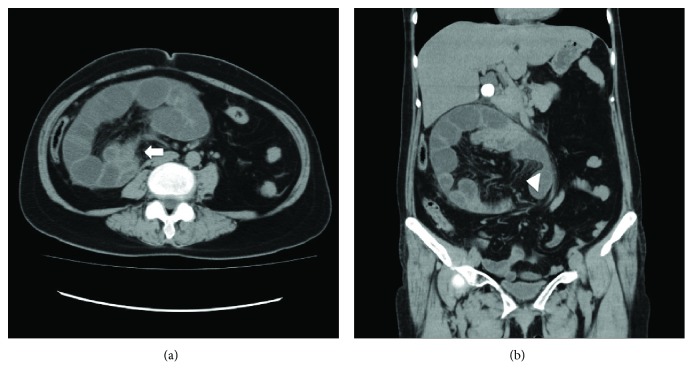
Abdominal CT (noncontrast-enhanced) findings in this patient. (a) Axial image showing that most of the small intestine was located in the right upper abdominal cavity and had a sac-like appearance (arrow). Ascites was observed within the sac. (b) Coronal image showing absence of the ligament of Treitz in the duodenum, along with a flattened inferior vena cava and distorted mesenteric veins (arrowhead). A 2 cm-sized gallstone was also observed.

A 56-year-old Japanese female was referred to our hospital for vomiting, nausea, and abdominal pain after excessive drinking and eating. Abdominal pain occurred 6 hours after her last meal, followed 2 hours later by frequent vomiting. On admission, she was alert, afebrile, 160 cm in height, and 57.2 kg in weight. Her blood pressure (BP) was 158/95 mmHg, her heart rate (HR) was 80/min, and her oxygen blood saturation (SpO2) was 100% (room air). Physically, she complained of pain in the swollen right upper quadrant but without muscular defense. Her previous history included an oophorectomy for ovarian cysts at age 26 years, conservative treatment for duodenal ulcer at age 36 years, lithotripsy for ureter stones at age 48 years, and conservative treatment for gallstones and cholecystitis at age 53 years. Laboratory data on admission included a white blood cell (WBC) count of 18,500/*μ*L, hemoglobin (Hb) concentration of 16.0 g/dL, platelet count of 308 × 10^3^/*μ*L, serum C-reactive protein (CRP) concentration of 1.06 mg/dL, and lactate dehydrogenase concentration of 204 U/L, with normal hepatic and renal function. Serum electrolyte levels were all within normal ranges. Abdominal computed tomography (CT) showed that most of the small intestine was located in the right upper abdominal cavity and had a sac-like appearance, without ligament of Treitz being present in the duodenum. CT of the vascular system showed a flattened inferior vena cava in association with edematous mesenterium and dilated mesenteric veins, findings suggesting a strangulated ileus (Figures 1(a) and 1(b)).

**Figure 2:**
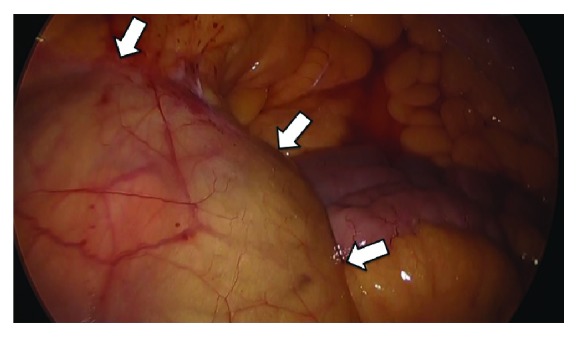
Photograph showing laparoscopic findings during surgery of the distended small intestine and ascites under the ascending mesocolon.

Emergency laparoscopic repair was performed. Under general anesthesia, a cut was made at the umbilicus, a 12 mm port was inserted using the open method, and 5 mm ports were inserted into the right and left lateral abdomen as well as the lower abdomen. Laparoscopic observation showed that the ascending and descending colons were in their normal positions, with the cecum in the right lower abdomen. However, ascites and distention of the small intestine were observed under the mesenteric membrane of the ascending colon. The duodenum showed a leftward and then a rightward bending, with most of the small intestine, except for a 1-meter-long section of the ileum in the ileocecal region, being packed within the ligament of Ladd, which formed a hernia sac (Figure 2). Although the small intestine was tightly adhesive within the hernia sac, careful peeling and incision release were performed without complications. The absence of severe ischemia within the incarcerated intestine precluded the need for intestinal resection. The patient's clinical course of postlaparoscopic repair was uneventful; she was able to drink water on day 2 and was discharged on day 9. Examination of an abdominal CT image of this patient taken at age 48 years showed a similar sac-like appearance, suggesting that this patient may have had intermittent PDH for 8 years. A follow-up CT scan after 2 months of surgical repair showed no abnormalities. At the time of writing, 3 years after surgical repair, the patient remains well without any recurrent symptoms or other complaints.

### 2.1. Literature Review

A survey of the ICHUSHI (Igaku Chuo Zasshi; the Japan Medical Abstract Society; http://www.jamas.or.jp) of patients in Japan who underwent laparoscopic repair of PDH during the period of 2002 to 2017, using the keywords PDH, laparoscopy, and adults (>18 years old), identified 26 such patients in 22 studies, all written in Japanese [14–35] and two more patients in two studies written in English [12, 36]. Including the present patient, a total of 29 patients were analyzed. The median age of these 29 patients was 52.3 years (range, 20–80 years), and the male to female ratio was 16/13. Eight patients had right-sided PDH while 21 had left-sided PDH. Their clinical features and laparoscopic repair results are shown in Table 1. Time from initial symptoms to diagnosis did not differ significantly between right and left-sided PDH. Emergency surgery was more frequent in patients with right-sided PDH. Additional procedures during laparoscopic repair were more frequent in patients with left-sided (6/21) than right-sided (1/8) PDH. Release of the hernia sac was more frequent in patients with right-sided PDH. Overall results were excellent, but a few patients with either right-sided or left-sided PDHs required additional postoperative care.

## 3. Discussion

PDHs are congenital mesocolic hernias caused by an abnormal rotation of the primitive midgut in embryonic life [11]. In cases of right PDH, the counterclockwise rotation of the midgut is arrested on the right side. As a result, the small bowel becomes trapped in a hernial sac formed by the peritoneum behind the colonic mesentery, where the cecum and ascending colon rotate anteriorly, while in left PDH, the small bowel invaginates into an avascular segment of the left mesocolon and is entrapped between the mesocolon and the posterior abdominal wall, forming the anterior wall of the hernia sac [4, 37].

This report describes an adult patient with right-sided PDH, which was successfully managed by laparoscopic repair. Since right-sided PDH is rare and is frequently accompanied by nonspecific clinical signs, it was reported that laparoscopy may be necessary to confirm the precise diagnosis [8]. However, in this case, we confirmed the diagnosis preoperatively with use of abdominal CT findings.

In the diagnosis of PDH, the symptoms and physical findings are vague and nonspecific, and plain abdominal radiographs and ultrasonography are also frequently nondiagnostic. Thus, the employment of abdominal CT is very helpful for the correct diagnosis. In fact, accurate preoperative diagnosis of PDH can be made by multidetector CT, which can detect the encapsulation of small bowel loops in the right midabdomen with looping of arterial and venous jejunal branches behind the superior mesenteric artery in right PDH and the encapsulation of bowel loops at or above the level of the ligament of Treitz with intermittent dilatation in left PDH [38].Contrast-enhanced CT may also be required for an accurate diagnosis of PDH [11].

PDH can be repaired by reduction of hernia contents and excision of the hernia sac, with or without intestinal derotation, thereby avoiding injury to the major mesenteric vessels juxtaposed to the hernial orifice. In the past, PDH was managed using laparoscopy or an open procedure [7, 8, 10]. However, a comparison of these two methods found that laparoscopy provided a more accurate preoperative diagnosis of PDH, as well as good postoperative outcomes, including shorter hospital stay and shorter times to first flatus and to first intake of a soft diet [10]. Good exposure of the operative field is critical to successful laparoscopic repair procedures [5]. Fundamentally, laparoscopic surgery does not differ for right- and left-sided PDH. However, right PDH is often associated with malrotation of the midgut and is sometimes complicated by strangulation, requiring division of the lateral attachments of the ascending colon with retraction to the left to reduce the size of the small bowel [39, 40]. By contrast, left-sided PDH can be reduced manually without much difficulty. More difficulties were therefore encountered in patients with right-sided PDH, as these patients require widening of the hernial orifice or division of the inferior mesenteric vein, as well as reduction of the invaginated intestine and suture closure of the hernial orifice [39, 41]. In some patients, the inferior mesenteric vein may have to be sacrificed to facilitate reduction of the hernia contents [42].

Our literature review of 29 Japanese patients matched a previously reported incidence of right (8/29; 27.6%) and left (21/29; 72.4%) PDH. The findings of early (13/29) and late (16/29) surgical interventions were also not significantly different from previous studies. It was noted that emergency surgery was more frequent in patients with right-sided PDH. In the review, we focused particularly on the patients who were technically difficult, who required additional surgical procedures during the laparoscopic repair of PDH. We found that such cases were more frequent in left-sided PDH, of which three experienced difficulties in peeling severe adhesions, one had a significantly distended bowel, and one required intestinal resection for necrosis. In addition, two patients showed involvement of the inferior mesenteric vein at the hernial orifice, requiring open laparotomy due to their high risk of damage to blood vessels. Recent advances in laparoscopic procedures is thought generally to have resulted in excellent outcomes of PDH repair, although several patients have required additional procedures.

In summary, early and correct diagnosis is essential to avoid intestinal obstruction and strangulation due to PDH in adult patients. Laparoscopic procedures can expedite diagnosis and repair by obtaining a clear view of anatomical malrotation. Currently, for any symptomatic cases with PDH, either right or left-sided, less invasive laparoscopic approach can be safely carried out without the risk of complications under the condition where sufficient skills and the suitable equipments are available. However, in technically difficult cases, it is recommended not to cling on laparoscopy but to switch to open surgery.

## Figures and Tables

**Table 1:** Characteristics of adult (>18 years) patients in Japan who underwent laparoscopic repair of right or left PDH.

	All	Right	Left
	29	8	21
Age (years), median (range)	52.3 (20–80)	56 (23–80)	50 (20–80)
Male/female ratio	16/13	4/4	12/9
Symptoms			
Abdominal pain	25	8	17
Nausea/vomiting	16	6	10
Abdominal distention	3	0	3
Constipation/other^∗^	2	0	2
Time from initial symptoms to diagnosis			
Early (<24 hours/1–7 days/7 days–3 months)	5/5/3	2/3/0	3/2/3
Late (3 months–1 year/>1 year/not described)	2/11/3	0/3/0	2/8/3
Timing of surgery			
Emergency	16	6	10
Elective	11	2	9
Not described	2	0	2
Surgical methods			
Total laparoscopy	22	7	15
Laparoscopy plus minilaparotomy	3	0	3
Laparotomy migration	4	1	3
Laparoscopic procedures			
Closure of hernial orifice	21	2	19
Release of hernia sac	8	6	2
Outcome			
Complete recovery	22	6	16
Postoperative conservative treatment for intestinal obstruction	5	1	4
Second operation needed	2	1	1

PDH: paraduodenal hernia. ^∗^Including one patient simultaneously diagnosed with inguinal hernia and PDH.
